# The effect of K-line classification in different cervical dynamic position on surgical outcomes in patients with ossification of the posterior longitudinal ligament after anterior controllable antedisplacement and fusion

**DOI:** 10.3389/fsurg.2022.987622

**Published:** 2022-09-23

**Authors:** Lin-Hui Han, Kai-Qiang Sun, Chen Yan, Jing-Chuan Sun, Jian-Gang Shi

**Affiliations:** ^1^ Department of Orthopedic Surgery, Spine Center, Changzheng Hospital, Second Military Medical University, Shanghai, China; ^2^ Department of Orthopedic Surgery, Naval Medical Center, Naval Medical University, Shanghai, China

**Keywords:** ACAF, k-line, OPLL, surgical outcome, classification

## Abstract

**Purpose:**

To investigate whether the K-line classification in different cervical dynamic position of patients with Ossification of the Posterior Longitudinal Ligament (OPLL) affects clinical outcome after Anterior Controllable Antedisplacement and Fusion (ACAF) surgery.

**Methods:**

A total of 93 patients who suffered from cervical spondylosis caused by OPLL underwent ACAF surgery between June 2015 and December 2017 in a single institution. Neutral, neck-flexed and neck-extended cervical radiographs were obtained from every patient. Subsequently they were classified into K-line (+) and K-line (−) with reference to the K-line classification criteria. Clinical outcomes were assessed by the JOA score, improvement rate (IR) and visual analogue scale (VAS). Radiological assessment included Cobb angle and occupation ratio (OR) of OPLL. Correlations between the long-term surgical outcomes and classification of K-line in different dynamic position were analyzed by one-way analysis of variance.

**Results:**

Significant improvements were shown in all postoperative clinical and radiographic assessments (*P *< 0.05). There were no differences in IR, Cobb angle and VAS among flexion K-line (−), flexion K-line (+), extension K-line (−) and extension K-line (+) at the 2-year follow-up (*P *> 0.05). However, the OR of extension K-line (−) (16.13% ± 11.58%) was higher than that of extension K-line (+) (9.00% ± 10.27%) and flexion K-line (+) subgroup (9.47% ± 9.97%) (*P *< 0.05).

**Conclusion:**

The ACAF procedure has shown satisfactory surgical outcomes in various K-line classifications in different dynamic position.

## Introduction

Ossification of the posterior longitudinal ligament (OPLL) is a disease in which the posterior longitudinal ligament is chronically ossified at various vertebral levels, with the more frequent occurrence in cervical spine, particularly at the C5 level (1–4). As ossification progresses, it will eventually cause spinal cord compression and produce corresponding clinical symptoms (5). The symptoms of the patients mainly include sensory and motor dysfunction of extremities and torso, and even paralysis and incontinence in severe cases (6, 7). Currently, surgical decompression is the mainstay treatment for OPLL.

Anterior Controllable Antedisplacement and Fusion (ACAF) is a novel technique for the treatment of OPLL, which could provide sufficient decompression and postoperative recovery rates and is less likely to have post-operative complications compared to conventional surgery (8–11). K-line, a new index that can evaluate the cervical alignment and the OPLL size in 1 parameter, was reported, which could predict outcome of OPLL posterior decompression surgery. Cervical laminoplasty resulted in poor outcomes for K-line (−) OPLL, and good outcomes for K-line (+) OPLL (12). However, some patients who were K-line (+) in the neutral position but K-line (−) in the neck-flexed position had poor clinical outcomes after cervical laminoplasty (13). There is a dynamic aspect of cervical myelopathy caused by OPLL (14). Thus, the K-line classification in different dynamic positions is of great relevance for clinical outcomes.

Whether the K-line classification in different dynamic positions affects the postoperative clinical outcome of ACAF surgery is not clear. Therefore, we conducted this retrospective study with a large sample size and long-term follow-up.

## Materials and methods

### Subjects

**Figure 1:**
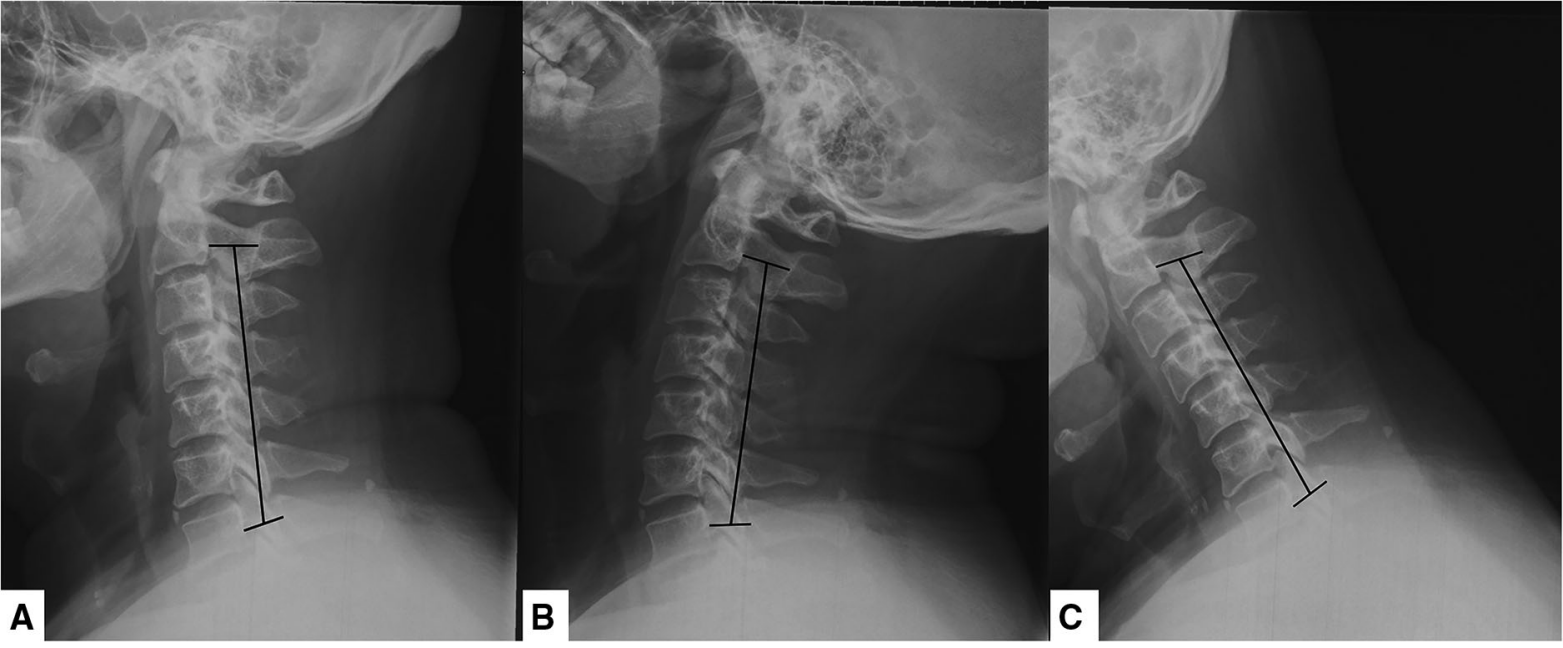
Cervical radiographs in different dynamic position. (**A**) Neck-neutral position; (**B**) neck-flexed position; (**C**) neck-extended position.

From June 2015 through December 2017, 93 patients with cervical myelopathy due to OPLL underwent ACAF in a single institution. All patients underwent ACAF by the same surgical team. Inclusion criteria included those patients with myelopathy caused by OPLL with a clear diagnosis based on clinical symptoms and imaging examination. Exclusion criteria included a history of spinal surgery, trauma, tumor, deformity, or infection and concurrent compressive lesions in the other spinal region. Patients with blurred and obscured imaging data were not included in the study. The 93 patients were classified into three groups based on the cervical radiographs in different dynamic position: the neutral position, the neck-flexed position and the neck-extended position. Afterwards, each group was divided into two subgroups based on K-line measurement of OPLL: K-line (+) subgroup and K-line (−) subgroup (Figure 1).

### Clinical evaluation and radiographic assessments

#### Clinical evaluation

Japanese Orthopedic Association (JOA) scores that was used to assess the degree of disability and improvement rate (IR) of neurologic function were investigated in all cases. Improvement rate was calculated as IR = (postoperative JOA score—preoperative JOA score/17-preoperative JOA score) ×100%. A Visual Analog Scale (VAS) was applied to measure neck pain and arm pain. Surgical outcome was defined by the IR as follows: excellent (IR ≥ 75%), good (75% > IR ≥ 50%), fair (50% > IR ≥ 25%), and poor (IR < 25%). The follow-up period was 2 years in all cases.

#### Radiologic evaluation

**Figure 2:**
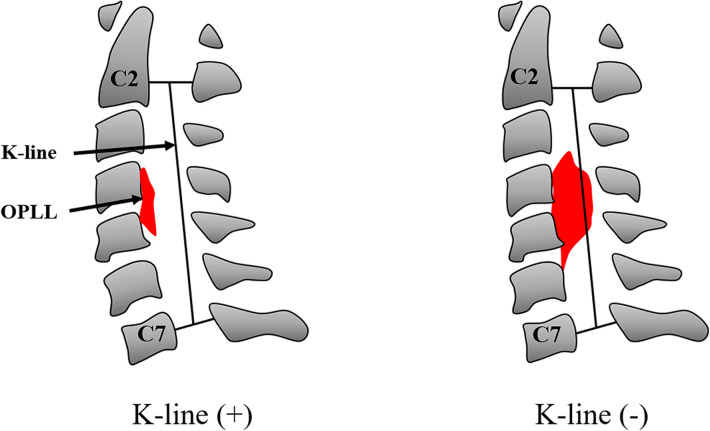
Schematic diagram of the K-line. The K-line is the line connecting the midpoints of the spinal canal at C2 and C7. The OPLL does not cross the K-line in K-line (+) group. The OPLL exceeds the K-line in the K-line (−) group. OPLL, ossification of the posterior longitudinal ligament.

All patients underwent plain radiography, computed tomography (CT), and three-dimensional reconstruction before and after surgery. The following parameters were investigated: (1) Cervical curvature is measured as the Cobb angle, the angle between a line parallel to the posterior aspect of the C2 vertebral body and that of the C7 body; (2) the rate of narrowing in the spinal canal is calculated by occupation ratio (OR). In the narrowest plane of the spinal canal, OR = (thickness of OPLL/spinal canal anteroposterior diameter) ×100%; (3) The K-line is the line connecting the midpoints of the spinal canal at C2 and C7. In the K-line (−) group, the OPLL exceeds the K-line, whereas in K-line (+) group, the ossified mass does not across the K-line (Figure 2).

### Operative procedures

**Figure 3:**
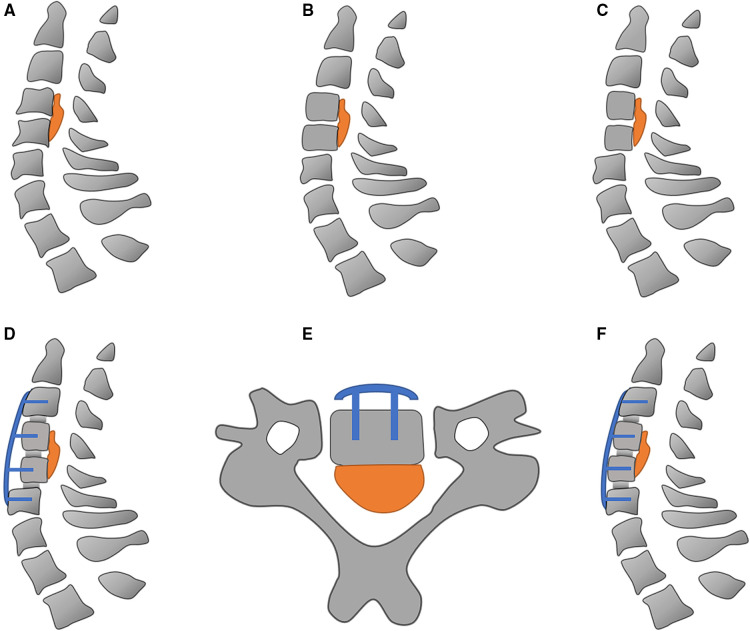
Schematic diagram of anterior controllable antedisplacement and fusion surgery. (**A**) The ossification of posterior longitudinal ligament at C4–C5; (**B**) conventional discectomy is performed in the involved levels; (**C**) resection of the anterior vertebral bodies of C4–C5; (**D**) installation of intervertebral cages, screws, and pre-bent anterior cervical plate; (**E**) creation of bilateral groove; (**F**) hoisting C4–C5 vertebrae.

(1) Anesthesia and exposure: After general anesthesia, supine position was adopted. The anterior structure of the cervical spine at the level of the OPLL was exposed and conventional discectomies were performed in the involved levels. (2) Resection of the anterior vertebral bodies of the vertebrae-OPLL complex (VOC): Then anterior parts of the vertebrae were cut according to the thickness of ossified ligament to allow for enough hoisting space. (3) Contralateral osteotomy: According to the expected decompression width, the contralateral osteotomy was performed using a high-speed drill. Then we used Kerrison rongeurs to remove the posterior vertebral wall on the bottom of the troughs. (4) Installation of the intervertebral cages and anterior cervical plate: Intervertebral carbon fiber cages were then placed into intervertebral spaces. The pre-bent anterior cervical plate and screws were then installed for temporary fixation on the middle vertebrae. (5) Isolation of VOC: An ipsilateral osteotomy was performed for complete isolation of the VOC. (6) Hoisting of VOC: The VOC were hoisted forward by tightening the screws mounted on the middle vertebrae (Figure 3).

### Statistical analysis

Data were analyzed using SPSS 26.0 statistical software. Statistical analysis was performed using the paired *t*-test, independent samples *t*-test and one-way analysis of variance (one-way ANOVA) as appropriate. *P* < 0.05 was considered statistically significant. Results are presented as the mean ± standard error.

## Results

### Clinicopathologic characteristics

Eligible for inclusion are 72 male patients and 21 female patients with a mean age of 57.56 years (range, 37–79 years). The mean JOA score for all patients increased from 11.69 ± 2.86 (range, 4–16) preoperatively to 15.96 ± 1.60 (range, 7–17) at the 2-year follow-up (*P* < 0.05). The mean pain intensity was 1.70 ± 1.91 (range, 0–7) on the VAS preoperatively, decreasing to 0.28 ± 0.67 (range, 0–3) at the 2-year follow-up (*P* < 0.05). The IR averaged 80.86% ± 31.13%. The mean cobb angle increased from 14.70° ± 8.68° (range, 0°–38.9°) at pre-operation to 20.26° ± 5.64° (range, 10°–32.6°) at the final follow-up (*P* < 0.05). The mean occupation ratio decreased from 53.98% ± 12.4% (range, 25.89%–79.05%) at pre-operation to 20.26% ± 5.64% (range, 10%–32.6%) at the final follow-up (*P* < 0.05). The spinal level of surgery involvement was C2–C4 for 1 patient, C2–C5 for 13 patients, C2–C6 for 10 patients, C2–C7 for 14 patients, C2–T1 for 1 patient, C2–T2 for 1 patient, C3–C5 for 1 patient, C3–C6 for 12 patients, C3–C7 for 18 patients, C4–C6 for 1 patient, C4–C7 for 18 patients, C4–T1 for 2 patients and C5–C7 for 1 patient. There were 26 patients with continuous type, 17 patients with segmental type, 31 patients with mixed type and 19 patients with local type. As shown in Table 1.

**Table 1:** Clinical preoperative and postoperative data of the patients.

Item	Value
Sex (*n*)	Male 72, female 21
Age (years)	57.56 ± 9.01 (37–79)
Japanese orthopaedic association score	
Before surgery	11.69 ± 2.86 (4–16)
2 years after surgery	15.96 ± 1.60 (7–17)*
IR (%)	80.86 ± 31.13 (−100 to 100)
VAS	
Before surgery	1.70 ± 1.91 (0–7)
2 years after surgery	0.28 ± 0.67 (0–3)*
Cobb angle (°)	
Before surgery	14.70 ± 8.68 (0–38.9)
2 years follow-up	20.26 ± 5.64 (10–32.6)*
Occupying ratio (%)	
Before surgery	53.98 ± 12.42 (25.89–79.05)
2 years follow-up	10.99 ± 11.07 (0–41.64)*
Surgery involvement (number of patients)	
C2–C4	1
C2–C5	13
C2–C6	10
C2–C7	14
C2–T1	1
C2–T2	1
C3–C5	1
C3–C6	12
C3–C7	18
C4–C6	1
C4–C7	18
C4–T1	2
C5–C7	1
Classification of OPLL (cases)	
Continuous type	26
Segmental type	17
Mixed type	31
Local type	19

Values are mean ± standard error (range).

* Statistically different from the data before surgery.

### Clinical data in two subgroups according to the K-line classification in neutral position

Of the 93 OPLL patients studied in our analysis, 64 were categorized as K-line (+) and 29 were categorized as K-line (−). The preoperative and postoperative clinical data are presented in Table 2. The mean IR was 79.61% ± 33.22% in the K-line (+) subgroup and 83.61% ± 26.26% in the K-line (−) subgroup. Therefore, there was no difference in post-operative neurologic improvement between the K-line (+) subgroup and K-line (−) subgroup in neutral position (*P* > 0.05). The Cobb angle, OR and VAS of both subgroups improved significantly after surgery compared to the final follow-up (*P* < 0.05). The Cobb angle was 20.64° ± 5.68° in the K-line (+) subgroup and 19.41° ± 5.53° in the K-line (−) subgroup (*P* > 0.05). The OR for the K-line (+) subgroup was 8.83% ± 10.29%, which was lower than the value for the K-line (−) subgroup 15.77% ± 11.39% (*P* < 0.05). The VAS was 0.36 ± 0.76 in the K-line (+) subgroup and 0.10 ± 0.31 in the K-line (−) subgroup (*P* < 0.05).

**Table 2:** Clinical data according to the K-line classification in neutral position.

Item	K-line (+) (*n* = 64)	K-line (−) (*n* = 29)	*P* value
Age (years)	57.6 ± 9.1	57.3 ± 9.0	0.878
Japanese orthopedic association score			
Before surgery	11.63 ± 2.84	11.83 ± 2.94	0.753
2 years after surgery	15.81 ± 1.81*	16.28 ± 0.96*	0.111
Improvement rate (%)	79.61 ± 33.22	83.61 ± 26.26	0.569
Cobb angle (°)			
Before surgery	16.30 ± 8.89	11.18 ± 7.13	0.008
2 years follow-up	20.64 ± 5.68*	19.41 ± 5.53*	0.330
Occupying ratio (%)			
Before surgery	51.71 ± 13.05	58.96 ± 9.27	0.003
2 years follow-up	8.83 ± 10.29*	15.77 ± 11.39*	0.004
Visual analog scale			
Before surgery	1.69 ± 1.85	1.72 ± 2.07	0.932
2 years after surgery	0.36 ± 0.76*	0.10 ± 0.31*	0.024

Values are mean ± standard error.

* Statistically different from the data before surgery.

### Clinical data in four subgroups according to the K-line classification in flexed and extended position

Flexion and extension x-rays of the cervical spine were obtained from every patient. They were then divided into different subgroups according to the K-line Classification. The preoperative and postoperative clinical data are shown in Table 3. All four subgroups of postoperative observations (JOA score, Cobb angle, OR and VAS) were better than these before surgery (*P* < 0.05). There were no differences in IR, Cobb angle and VAS at the 2 years follow-up among the four subgroups (*P* > 0.05). However, there was a statistical variance among the OR. The OR of extension K-line (−) subgroup (16.13% ± 11.58%) is higher than that of extension K-line (+) subgroup (9.00% ± 10.27%) and flexion K-line (+) subgroup (9.47% ± 9.97%) (*P* < 0.05).

**Table 3:** Clinical data according to the K-line classification in the cervical dynamic position.

Item	Flexion K-line (+)	Flexion K-line (−)	Extension K-line (+)	Extension K-line (−)	*P* value
Number of cases (*n*)	35	59	67	26	
JOA score					
Before surgery	11.37 ± 2.70	11.88 ± 2.96	11.75 ± 2.84	11.54 ± 2.97	
2 years after surgery	15.54 ± 2.20^a^	16.21 ± 1.04^a^	15.84 ± 1.78^a^	16.27 ± 0.96^a^	
Improvement rate (%)	80.02 ± 26.10	81.36 ± 34.02	79.03 ± 34.09	85.56 ± 21.62	0.835
Cobb angle (°)					
Before surgery	17.21 ± 9.61	13.19 ± 7.77	16.23 ± 8.86	10.79 ± 6.90	
2 years follow-up	21.83 ± 5.99^a^	19.31 ± 5.24^a^	20.50 ± 5.60^a^	19.63 ± 5.79^a^	0.183
Occupying ratio (%)					
Before surgery	52.67 ± 15.87	54.76 ± 9.85	51.55 ± 12.86	60.22 ± 8.63	
2 years follow-up	9.47 ± 9.97^a^^,^*	11.91 ± 11.67^a^	9.00 ± 10.27^a^^,^*	16.13 ± 11.58^a^	0.03
Visual analog scale					
Before surgery	1.63 ± 1.90	1.74 ± 1.93	1.66 ± 1.84	1.81 ± 2.12	
2 years after surgery	0.40 ± 0.85^a^	0.21 ± 0.52^a^	0.34 ± 0.75^a^	0.12 ± 0.33^a^	0.256

JOA, Japanese orthopedic association.

^a^ Statistically different from the data before surgery.

* Statistically different from the Extension K-line (−) subgroup.

## Discussion

OPLL is more common in Asia, and the formation of ossification can cause compression of the spinal cord (15, 16). For asymptomatic OPLL patients, conservative treatment is the preferred option. If the patient has progressive myelopathy due to compression of the spinal cord by ossification, surgery will be necessary (17, 18). There are two categories of surgery: anterior and posterior decompression surgery (19). Patients with K-line (−) have difficulty in achieving satisfactory spinal drift and optimal neurological recovery after posterior decompression (20). Anterior approaches are superior to posterior techniques for >60% OPLL canal occupancy with cervical kyphosis (21, 22). When performing an anterior approach, however, the direct resection of the ossification with a limited surgical field of view makes it much riskier and more complicated (23). The OPLL is not directly removed during the ACAF surgery, an innovative technique for the treatment of OPLL. By resection of the anterior vertebral bodies of the vertebrae, the vertebral ossification complex (VOC) is hoisted and the spinal cord and nerve root then receive direct decompression (8). According to previous study, ACAF is more effective in treatment of multilevel severe OPLL compared with laminoplasty (9).

The ACAF technique has a favorable effect on both short and long term post-operative neurological improvement in patients. One-year follow-up data showed IR of 60.1% ± 9.2% for the ACAF technique (24). In the present study, 2-year follow-up indicated the IR was 80.86% ± 31.13%.

Dynamic factors are not associated with the clinical outcome of ACAF surgery. Patients with various K-line classifications, measured in different dynamic position, obtained ideal treatment results. Recent reports have described poor neurological improvement in patients with K-line (−) OPLL in the neck-flexed position after cervical laminoplasty (13, 25). In comparison, the ACAF technique was very effective in patients with K-line (−) in the neck-flexed position, with a mean improvement rate of 81.36% ± 34.02%. Meanwhile, the ACAF technique was found to be effective in treating patients with OPLL under other classifications [flexion K-line (+), extension K-line (+) and extension K-line (−)] in the current study. Statistical analysis showed no difference in the clinical outcome of ACAF among these four subgroups (*P* > 0.05). The classification of K-line is influenced by both the alignment of the cervical spine and the size of the ossification. Both can cause the ossification to exceed the K-line and meet the K-line (−) criterion. The ACAF procedure is able to restore the physiological curvature of the cervical spine and hoist VOC by means of a pre-bent titanium plate, allowing the K-line to move backwards. Cervical Cobb angle has been changed from 14.70° ± 8.68° preoperatively to 20.26° ± 5.64° 2 years after surgery (*P* < 0.05). OR has been changed from 53.98% ± 12.42% preoperatively to 10.99% ± 11.07% 2 years after surgery (*P* < 0.05). ACAF surgery solves above problems and therefore provides maximum decompression of the spinal cord, resulting in a favorable outcome. The change of cervical curvature and the relative backward movement of ossification may re-induce spinal cord compression in the neck-flexed position after cervical laminoplasty (13). ACAF surgery through discectomy, intervertebral carbon fiber cages implantation and pre-bend plate fixation makes the structure of the surgical site relatively stable. The ossification will not move with flexion or extension of the neck.

The postoperative OR in the neck-extended K-line (−) subgroup was higher than the other two K-line (+) subgroups which were measured in cervical dynamic position. This may be explained by the thicker ossification in extension K-line (−) subgroup, which made it necessary to resect more of the anterior vertebral bodies of the vertebrae to provide more hoisting space. However, if too much bone is removed, even the shortest cervical screws can penetrate the remaining vertebrae, causing damage to the spinal cord (26). Consequently, the postoperative OR for these patients was higher.

The relatively small sample size is one of the limitations of our study, especially in extension K-line (−) subgroup. This may be associated with the fact that the lesions are theoretically most severe in these patients. Second, this study is a single institution and retrospective study inherently involved selection bias. Multi-center investigation is required for future studies. The relatively short follow-up period is another limitation of this study, although the 2-year follow-up is already the longest follow-up period in ACAF surgery studies. Control group which underwent posterior surgery from the same institution is needed. In the further study, we will set up posterior surgery as a control to investigate the effect of the K-line classification in different dynamic positions on the outcome of the procedure. Also, the sample size will be expanded and the follow-up period will be increased.

## Conclusion

The ACAF surgery has excellent results in treating OPLL under each K-line classification in different dynamic positions. ACAF surgery has wide range of applicability for the treatment of OPLL.

## Data Availability

The raw data supporting the conclusions of this article will be made available by the authors, without undue reservation.
